# Vital Pulp Therapy—Current Progress of Dental Pulp Regeneration and Revascularization

**DOI:** 10.1155/2010/856087

**Published:** 2010-04-28

**Authors:** Weibo Zhang, Pamela C. Yelick

**Affiliations:** Division of Craniofacial and Molecular Genetics, Department of Oral and Maxillofacial Pathology, Tufts University, 136 Harrison Avenue, Room M824, Boston, MA 02111, USA

## Abstract

Pulp vitality is extremely important for the tooth viability, since it provides nutrition and acts as biosensor to detect pathogenic stimuli. In the dental clinic, most dental pulp infections are irreversible due to its anatomical position and organization. It is difficult for the body to eliminate the infection, which subsequently persists and worsens. The widely used strategy currently in the clinic is to partly or fully remove the contaminated pulp tissue, and fill and seal the void space with synthetic material. Over time, the pulpless tooth, now lacking proper blood supply and nervous system, becomes more vulnerable to injury. Recently, potential for successful pulp regeneration and revascularization therapies is increasing due to accumulated knowledge of stem cells, especially dental pulp stem cells. This paper will review current progress and feasible strategies for dental pulp regeneration and revascularization.

## 1. Introduction

Endodontic therapy, also known as root canal treatment, is one of the most commonly used techniques in dental clinics. Endodontic therapy is a procedure for removing contaminated or injured dental tissue, refilling, and sealing off the created void with synthetic material to eliminate future contamination. With advancements in antibiotic therapies, dental materials, and endodontic technology, the success rate of endodontic therapy has increased dramatically over the past decade [1]. The outcomes of certain cases which previously were considered intricate or of uncertain result, such as secondary root canal treatment, now achieve high levels of clinical success [2, 3]. That is to say, endodontically treated teeth now can maintain their function, for prolonged periods of time without a living pulp. 

Current endodontic procedures replace the vital pulp with synthetic materials, rather than living tissue. Extruded endodontic materials can cause a foreign body reaction [4]. Pulpless teeth lose their ability to sense environmental changes, making the progression of caries unnoticeable by patients. Another advantage of maintained dental pulp vitality is to maintain the capacity for limited dentin regeneration. Reparative dentin formation is particularly important for immature permanent teeth, because of their incomplete apical and dentinal wall development. The structural integrity of endodontically treated teeth may also be undermined if they are not properly restored, making them more vulnerable to masticatory forces [5]. In terms of aesthetics, endodontic therapy can often result in discoloration of the tooth crown, mainly due to staining from endodontic filling material [6]. Maintaining the vital pulp also helps reduce the occurrence of apical periodontitis by blocking bacterial infections [7, 8]. Based on these issues and concerns, the ability to maintain or renew dental pulp vitality would be preferable to current endodontic treatments [9].

In this paper, we will discuss the current status and future prospects for successful dental pulp regeneration and revascularization therapies.

## 2. The Biology of Dental Pulp

Dentin, one of the main mineralized tissue components of teeth, is a hard tissue with dentinal tubules penetrating throughout the entire thickness. The dental pulp is a heterogeneous soft tissue located in the center of teeth, which contains a variety of cell types and extracellular matrix molecules. Both dentin and the pulp are derived from neural crest cells. Because of their close relationship, especially during embryonic stages of tooth development, it is difficult to discuss these two types of tissues separately.

The primary function of pulp is to produce dentin, including primary dentin during early tooth development, secondary dentin throughout the entire life span of the tooth, and tertiary dentin under pathogenic stimuli. Odontoblasts, a layer of cells lining the periphery of the pulp at the inner dentin surface, are the specialized cell type capable of synthesizing dentin. The dental pulp is a highly vascularized tissue with abundant myelinated and unmyelinated nerves. This property correlates with the other two main functions of the dental pulp, which are to provide nutrition to dentin, and to function as a biosensor to detect unhealthy stimuli [10]. 

Anatomically, the dental pulp is almost fully encapsulated by hard dentin. The only connection between the dental pulp and the surrounding tissue is through the tiny root apexes. All of the main blood vessels and lymph drainages of dental pulp pass though the tooth root apexes, which make the apex the main pathway for tooth nutrition and waste exchange. In some teeth, there are also much smaller openings of lateral canals, located near the apical foramen. This limited accessibility and unyielding environment of the dental pulp makes it difficult to eliminate inflammation, once it has occurred. 

Injured dental pulp has limited potential for self-recovery. If the stimuli are mild or progress slowly, such as occur in the cases of mild caries, moderate attrition, erosion, or superficial fracture, odontoblasts can usually survive and continue to produce the dentin barrier beneath the injury, allowing the underlying soft pulp tissue to retain its function. The essential strategy under these situations is to protect the remaining odontoblasts. When the stimuli are strong and/or rapidly progressing, such as occur in deep dentin caries, severe abrasion, and fracture, the primary odontoblasts will be destroyed. In these cases, the postmitotic terminally differentiated odontoblasts lack the ability to proliferate to replace injured odontoblasts, or to produce new dentin. Under these circumstances, undifferentiated mesenchymal cells within the dental pulp can differentiate into odontoblasts and secrete reparative dentin. Under these circumstances, undifferentiated mesenchymal cells within the dental pulp can differentiate into odontoblasts and secrete reparative dentin [11]. These descriptions fit the profile of stem cells. Undifferentiated mesenchymal cells within the pulp also have the potential to differentiate into other cell types, including fibroblasts, to repair the damaged soft pulp tissue. The ability to stimulate the stem cell differentiate into odontoblasts-like cells, rather than fibroblasts, is critical in dentin repair.

## 3. Regeneration and Revascularization of Dental Pulp

Although pulp regeneration and revascularization is not essential, due to the fact that the pulpless tooth can survive for a long time after a successful endodontic treatment, maintaining the vitality of dental pulp provides many benefits. Generally speaking, depending on whether any vital dental pulp is still left or not, there are two main approaches for dental pulp regeneration and revascularization, either vital pulp therapy, or whole pulp regeneration.

### 3.1. Vital Pulp Therapy

**Figure 1:**
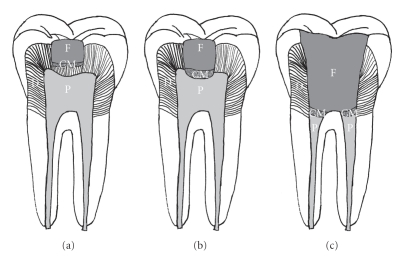
Vital pulp therapy. (a) Indirect pulp capping; (b) direct pulp capping; (c) pulpotomy. (D = Dentin; P = Pulp; CM = Capping material; F = Filling).

The aims of vital pulp therapy are to maintain the vitality of the dental pulp, and to stimulate the remaining pulp to regenerate the dental-pulp complex. Clinically, vital pulp therapy can be divided into two main groups: indirect pulp capping and direct pulp capping/pulpotomy (Figure 1). Indirect pulp capping is achieved by applying a protective agent on the thin layer of dentin remaining over a nearly exposed pulp, in order to allow the underlying dental pulp to recover [12]. In contrast, direct pulp capping is the strategy where a protective agent is placed directly on the exposed pulp to protect the underlying pulp from further injury, and to allow the dentin-pulp complex to regenerate [13]. When dental pulp exposure is large, or the pulp is infected, all of the coronal pulp must be removed, and direct pulp capping will subsequently be performed adjacent to the root pulp. This method is called pulpotomy [14]. After pulpotomy treatment, the dental pulp within the root canal can be preserved, and the roots of immature teeth can continue to grow [15, 16].

There are two main strategies to achieve a successful vital pulp therapy, to reduce further damage of existing odontoblasts, and to induce the differentiation of new odontoblasts. A successful vital pulp treatment requires a good sealant against bacteria, no severe inflammatory reactions, and stable haemodynamic within the pulp [17]. The ideal prognosis also includes the formation of a continuous dentin bridge at the pulp-dentin border. This newly formed dentin is comparatively less mineralized, and softer, as it contains more organic material. Still, it helps to block stimuli from the outside and thus to protect the pulp vitality. However, the formation of osteodentin, dentin with an osteotypic appearance, and scar-like soft tissue is also regarded as successful healing, although osteotypic hard tissue cannot provide the necessary barrier effect to protect the pulp from exogenous destructive stimuli [18]. 

Two separate responses can significantly influence the successful outcomes of pulp capping therapies. The first is the response to the operative procedure, and the second is the reaction to the restorative modalities. As a basic requisite for successful healing, sterile principles should be applied during all restoration procedures. It is necessary to relieve the inflammatory reaction of the irritated pulp and to control the bleeding before restoring a tooth with a permanent material. A layer of restorative material can be applied on top of the wound after removing contaminated dental tissue and control the contamination. The restorative material should not only offer the dentin-pulp complex a relative stable environment, but also support the regeneration of dentin-pulp complex. In this regard, treatment modalities should be able to induce the differentiation of odontoblasts. The most commonly used restorative materials include calcium hydroxide, adhesive resin-based composites systems, glass-ionomer materials, and zinc oxide eugenol (ZOE). Those inorganic restorative materials are unable to induce cell differentiation. For this reason, the delivery of growth factors and/or use of growth factor-embedded materials are good complements for vital pulp capping. 

Growth factors are an extensive group of proteins which can induce cellular proliferation and differentiation by binding to receptors on the cell surface. A variety of growth factors have successfully been used for dentin-pulp complex regeneration, including Transforming Growth Factors (TGFs) [19], Bone morphogenetic proteins (BMPs) [20], Platelet-derived growth factor (PDGF) [21], Insulin-like growth factor (IGF) [22], and fibroblast growth factors (FGFs) [23]. Among those, BMP-2 [24], BMP-4 [25], BMP-7 [26] have been shown to direct pulp progenitor/stem cell differentiation into odontoblasts and result in dentin formation, making the BMP family the most likely candidate for dental clinic applications. Promising results include an autogenous transplantation of recombinant human BMP2-treated porcine dental pulp to the amputated pulp, resulting in the formation of reparative dentin and odontoblast-like cells with long processes attached to newly formed osteodentin, as were observed after 4 weeks [27].

Some natural materials are used for pulp capping because they contain growth factors. The most commonly used one is dentin, because bioactive molecules released from dentin can promote dentinogenesis. It has been described that odontoblast-like cells and reparative dentin can be observed when EDTA-demineralized dentin was used as capping material [28]. Enamel matrix derivative (EMD) is also capable of inducing dentin formation when applied to the dentin-pulp complex [29], although the mechanism for this repair has not yet been clarified. One possibility is that amelogenins present within the enamel extracellular matrix may take part in, or direct dentinogenesis. However, growth factor delivery alone cannot work effectively in the cases exhibiting inflamed pulp tissue [30]. 

Vital pulp capping provides the advantage of maintaining the vitality of the dental pulp. However, dental pulp tissue is easily irritated, and the irritants are difficult to remove due to the limited accessibility to the dental pulp. These facts restrict the self-recovery potential of the dental pulp. So, vital pulp therapy is only recommended for teeth that are asymptomatic, or which exhibit only minimal inflammatory response symptoms.

### 3.2. Whole Pulp Regeneration

Because vital pulp therapy outcomes are difficult to predict, endodontic treatments are widely used in the clinics currently. Whole pulp regeneration should be considered if the pulp has to be removed completely. Until now, there is no successful report of whole pulp regeneration in the clinic. For functional pulp regeneration, two issues must be considered: (1) how to induce odontoblast differentiation; (2) how to revascularize the regenerated dental pulp. The presence of differentiated odontoblasts lining the inner wall of the pulp chamber and root canal can facilitate repair of the functional dentin-pulp complex. However, when odontoblast differentiation occurs throughout the regenerated pulp, pulp stone formation may occur, which can block the blood supply, which is supplies only from the narrow apical end of the tooth, and cause pulp necrosis. Stem-cell-based tissue engineering and autogenous tooth implantation provide potential strategies for successful pulp regeneration.

#### 3.2.1. Stem-Cell-Based Tissue Engineering of Dental Pulp

The concept of “tissue engineering” was conceived by Langer and Vacanti in the early 1990s to describe the technique for biological tissue regeneration [31]. Cells, molecular signals, and scaffolds are the three main components of tissue engineering.

**Cell Source**

The most promising cell sources for tissue engineering are stem cells. A stem cell is an undifferentiated cell, which has the potential to proliferate and generate progenitor cells that can differentiate into specialized cells throughout postnatal life [32]. Although there are unsolved questions and usage limitations regarding stem cells, stem cell research remains one of the most active of academic fields. Based on their origin, there are two main types of stem cells-embryonic stem cells (ES cells) and postnatal or adult stem cells (AS cells).

Embryonic stem cells are stem cells derived from the inner cell mass of an early, preimplantation stage embryo known as a blastocyst. ES cells are pluripotent cells, which means that they can give rise to all differentiated cell types derived from all three germ layers. There are limited numbers of publications about ES cells in pulp regeneration, due to the restricted policies regarding ES cell research over the past few years. The possible donor-host rejection of human ES cells is another concern [33]. Mouse ES cells, mixed together with hydroxyapatite/tricalcium phosphate (HA/TCP) powders, have been transplanted into tooth sockets of Sprague-Dawley rats. Although immature bone tissue was observed after 12 weeks, no dentin or pulp-like tissue was found in the implants [34].

Tooth buds are another source of cells that have been used for dental tissue regeneration [35]. Tooth buds contain both dental epithelial and mesenchymal cells, and several groups have reported the formation of bioengineered teeth with anatomically correct tooth-crown shape, and enamel, dentin, and pulp tissues, using dental cell reaggregated tooth bud cells [36–38]. Similar results were achieved by replacing dental mesenchymal cells with mesenchymal cells obtained from other sources, including embryonic stem cells, neural stem cells, and adult bone-marrow-derived cells [39]. Generally speaking, the most successful regenerated tooth structures were obtained using cells from mouse embryonic tooth buds harvested E11~14.5. Other reports indicated the formation of recognizable tooth structures, containing organized enamel, dentin, and a well-defined tooth pulp, by seeding dissociated postnatal tooth bud derived epithelial and mesenchymal cells onto biodegradable materials [40–44]. Unlike the embryonic tooth bud cells, the postnatal tooth bud cells organized within the scaffold to form multiple, small individual tooth crown-like structures, although aberrant cusp morphology was also observed. These studies of tooth bud cell characterizations for whole tooth engineering provide useful information about the mechanism of tooth regeneration. However, without identified and suitable autologous human tooth buds, it will be difficult to develop widely applicable tooth regeneration strategies for humans.

AS cells are the self-renewable progenitor cells residing within most differentiated tissues and organs. AS cells are thought to migrate to the area of injury and differentiate into specific cell types to facilitate repair of the damaged tissues. Adult stem cells are found in almost all kinds of tissues, and have also been isolated from a variety of dental tissues, including dental pulp [45, 46], periapical follicle [47, 48], and periodontal ligament [49, 50]. The dental pulp stem cells (DPSCs) are clonogenic and proliferate rapidly. DPSCs can differentiate to odontoblasts, which makes them the most promising candidate for dentin-pulp complex regeneration. After being transplanted into immunocompromised mice, these cells generated mineralized dentin with highly organized tubular structures. Histological analyses revealed a well-defined layer of odontoblast-like cells, with characteristic processes extending into tubular structures within the regenerated dentin, and a highly vascularized pulp tissue center. The orientation of the collagen fibers within the dentin was perpendicular to the odontoblasts-like cell layer, similar to the naturally formed dentin [45, 46].

DPSCs, similar to other types of adult stem cells, have self-renewable ability and multilineage differentiation potential, including the ability to differentiate into neurons of the peripheral nervous system [51–53]. Dental pulp is derived from migrating neural crest cells, suggesting that DPSCs might be an appropriate candidate for nerve regeneration [54]. Based on cellular morphology and expression of early neuronal markers, DPSCs were capable of neuronal cell differentiation when cultured in the neurogenic medium in vitro [51, 52]. When transplanted into the mesencephalon of embryonic chicken embryo, DPSCs exhibited a neuronal morphology, with positive expression of neuronal markers [55]. Regenerated nerves with GFP-positive cells were observed when GFP-positive DPSCs were transplanted into a rat facial nerve gap in vivo [56]. In addition, DPSCs can produce an array of neurotrophic factors, including nerve growth factor, brain-derived neurotrophic factor, and glial cell line-derived neurotrophic factor, which support the idea that DPSCs are useful for nerve regeneration [57]. Nosrat et al. reported that dental pulp tissue grafted into hemisected spinal cord increased the number of surviving motoneurons, consistent with the idea that dental pulp-derived neurotrophic factors may play an important role in orchestrating the dental pulp innervations [58].

Investigations conducted by About's group revealed that human pulp fibroblasts from third molars express two important pro-angiogenic factors, vascular endothelial growth factor (VEGF) and basic fibroblast growth factor (FGF-2). The expression pattern of both angiogenic growth factors was very rapid and corresponded well to the pathological changes in the pulp following injury [59]. VEGF and FGF-2 both play essential roles in neovascularization of damaged tissue [60, 61]. The expression of specific antigens for endothelial cells, including von-Willebrand, CD31, and angiotensin-converting enzyme, was observed in human DPSCs population, suggesting an angiogenic potential for DPSCs [62]. Retroviral-GFP labeled DPSCs injected intramyocardially into myocardial infarcted nude rats revealed increased angiogenesis at the injury site, but no GFP^+^ endothelial, smooth muscle, or cardiac muscle cells were detected within the infarct [63]. Another study indicated that CD31^−^/CD146^−^ side population (SP) cells from dental pulp stem cells expressed CD34 and vascular endothelial growth factor-2 (VEGFR2)/Flk1, similar to endothelial progenitor cells (EPCs). In models of mouse hind limb ischemia, local transplantation of this DPSCs SP fraction resulted in successful engraftment and increased blood flow, including high density capillary formation. The transplanted cells were in close proximity to the newly formed vasculature and expressed several proangiogenic factors [64]. Further studies from the same group demonstrated that CD31^−^/CD146^−^  SP DPSCs could completely regenerate pulp tissue with capillaries and neuronal cells within 14 days [65].

DPSCs, harvested from deciduous teeth, were named stem cells from human exfoliated deciduous teeth (SHED) [66]. Similar to their adult tooth counterpart, SHED also exhibit multilineage differentiation potential including neurogenic potential, can support innervations, and are able to form dentin-pulp complex in vivo. SHED have been seeded onto a synthetic D,D-L,L-polylactic acid (PLGA) scaffolds and implanted into cleaned and reshaped mini pig teeth. Ultrastructural investigations demonstrated the adherence of SHED within the pulp constructs, suggesting the potential use of SHED-based implants for vital pulp regeneration in endodontically treated teeth [67]. SHED were also demonstrated to differentiate into odontoblast-like, and endothelial-like cells, when seeded onto tooth slices containing a poly-L-lactic acid (PLLA) polymer scaffold packed pulp cavity [68]. SHED formed a microvascular network, a prerequisite for the successful engineering of most tissues and organs [69].

Recently, another population of DSC, stem cells from the apical papilla (SCAP) of incompletely developed teeth, has been identified. Evidence for this unique DSC population is based on the observation that tooth root formation was demonstrated to continue in some immature teeth, following endodontic treatment [70]. SCAP, like DPSCs and SHED, can also differentiate into odontoblast-like cells and produce dentin-pulp complex in vivo [71, 72]. Since the apical papilla is located at the tip of root and receives blood supply from surrounding tissues, SCAP may survive after pulp necrosis or endodontic treatment and continue to produce root dentin [48].

**Scaffolds**

Another essential component of tissue engineering is scaffolds. An appropriate scaffolding material must support the attachment, proliferation, and differentiation of seeded stem cells. For dental pulp regeneration, the ideal scaffold should also support vascularization and innervations of pulp tissue. Most DPSCs studies have focused on the regeneration of the dentin-pulp complex [66], revealing for the most part, poorly organized dentin-pulp complex-like structures with random shapes and orientations. In contrast, for clinical applications, the regenerated tissue needs to be highly organized. A regenerated highly vascularized soft tissue core with surrounding hard tissue seal would result in the best prognosis. Other studies have focused on soft tissue pulp regeneration. Mooney et al. reported that human DPSCs seeded onto a 3D PGA matrix and grown in vitro formed new tissue with a cellularity similar to that of native pulp [73]. Further studies from the same group showed limited cell proliferation on collagen gels, and no cell proliferation on alginate scaffolds [74]. Since the dentin surrounding the pulp chamber can provide sufficient structural support, physical support from the scaffold is not necessary. Some soft 3D scaffold materials, including injectable hydrogels, may therefore be suitable for pulp regeneration. A self-assembling peptide-amphiphile (PA) hydrogel encapsulated with DPSCs or SHED, cultured in vitro in osteogenic medium, was demonstrated to express osteoblast markers, and deposit mineral, while SHED showed no sign of hard tissue formation, but rather collagen production [75].

**Growth Factors**

The third important factor for tissue engineering is to select appropriate growth factors. As mentioned earlier, morphogens such as BMPs can induce DPSCs to differentiate into odontoblast-like cells. How to deliver the growth factors effectively is one of the main challenges we are facing now. Direct application of growth factors often results in only temporary release. The limited half-life and unstable release of growth factors are unfavorable for new tissue formation. As compared to protein therapy, gene therapy is an alternative approach that may overcome these disadvantages. Mouse dental papilla cells transfected with growth/differentiation factor 11 (*Gdf11*) were demonstrated to express dentin sialoprotein (Dsp) [76]; Osteo-dentin formation during pulpal wound healing was observed in dog teeth in vivo after Gdf11 electroporation. The same group also used *Gdf11 *ultrasound-mediated gene delivery using microbubbles, demonstrating complete reparative dentin formation in animal model in vivo [77]. The effectiveness of this kind of in vivo gene therapy highly depends on the vitality of the remaining dental pulp cells. Ex vivo gene therapy, involving the transfer of in vitro transfected cells back in vivo, may provide a better solution. The Nakashima group also proved that the transplanted *Gdf11*-electrotransfected pulp cell pellet stimulated reparative dentin formation [78].

A tooth slice model has been successfully used to analyze repair of the dentin-pulp complex [19, 20]. This model has recently been modified to study dental pulp regeneration [68, 79]. The basic approach for this model is to fill the center void of the tooth slice with a biodegradable scaffold, followed by subsequent seeding with dental stem cells. When implanted in vivo, the seeded dental stem cells were able to differentiate into odontoblasts and endothelial-like cells. However, it is not clear whether this thin tooth slice model can successfully be adapted to regenerate full sized, vascularized dental pulp tissues for clinical applications. Limited blood supply is a major concern for de novo pulp regeneration. In a more recent study, a modified model was developed, which used a human tooth root fragment (6-7 mm long) with an enlarged root canal (1.0–1.25 mm wide), with one end sealed to mimic a natural tooth root [80]. Dental stem cells were seeded onto a poly-D,L,-lactide and glycolide (PLG) scaffold, which was then inserted into the fabricated tooth root. The cell-seeded tooth fragments were transplanted subcutaneously and harvested after three to four months. Analyses of the harvested implants revealed the formation of well-vascularized soft tissue in the root canal space, and a continuous layer of dentin-like tissue lined with odontoblast-like cells. These results verified the feasibility of the root fragment model for pulp regeneration. However, it is recognized that the subcutaneous environment is quite different from that of alveolar bone. It remains to be seen whether this model will be successful when implanted into the jaw bone of sheep and minipig animal models.

The potential for pulp-tissue regeneration from implanted stem cells has yet to be tested in clinical trials. Extensive clinical trials to evaluate efficacy and safety are required before it is likely that the Food and Drug Administration (FDA) will approve regenerative endodontic procedures using stem cells in humans [81].

#### 3.2.2. Autogenous Tooth Transplantation

Many methods have been developed to fill edentulous spaces caused by tooth loss and/or genetic tooth agenesis. Dental implants and tooth transplantations are the two most commonly used techniques. Dental implant success highly relies on clinician skill, the quality and quantity of the bone available at the implant site, and also the patient's oral hygiene and overall health. The general consensus of opinion is that implants carry a success rate of around 95% over 15 years [82–84], which makes dental implants the most popular method for replacing a missing tooth at the present time. However, as compared to dental implants, tooth transplantation is much faster, and less expensive. Because allogenic tooth transplantation can cause immunological rejection and disease transmission, it is mainly used for basic research [85]. Autologous tooth transplantation using available third molar wisdom teeth is an economically feasible clinical therapy, and teeth exhibiting two-thirds root formation are considered to be ideal for reimplantation [86]. Another advantage of tooth transplantation is the possibility for pulp regeneration. Pulp regeneration, revascularization, and reinnervation have been observed in both experimental animal studies [85], and also in humans studies [87]. During the surgical procedure of tooth removal, the pulp and periodontal ligament are ruptured, and the avulsed tooth often undergoes pulp necrosis and infection. Revascularization of the necrotic pulp is possible, but the apex opening needs to be more than 1.1 mm, and the tooth needs to be replanted within 45 minutes [88]. Rapid revascularization can prevent infection and support the continuous development of the tooth root. Although the clinical potential for autologous tooth transplantation has been confirmed, the limited supply of available donor teeth restricts the practical use of this technique. 

For successful pulp regeneration, revascularization is necessary. A blood clot needs to be produced to achieve possible root-canal revascularization. For endodontic treatment, it is recommended to create a blood clot after the contaminated tissue removal and infection control treatment [89]. The mechanism of how a blood clot benefits the root-canal revascularization is not entirely clear, although one possible reason is that SCAP cells from the apical papilla may migrate into the root canal and produce dentin-pulp complex-like tissue. Another possible mechanism is the delivery of abundant growth factors within the blood clot, such as platelet-derived growth factor. Finally, the blood clot may also act as a natural scaffold for cell attachment, proliferation, and differentiation.

## 4. Prospects for Pulp Regeneration

The ultimate goal of both vital pulp capping and endodontic treatment is to completely regenerate the dentin-pulp complex, both structurally and functionally. Ideally, the goal is to regenerate a vital dental pulp covered with dentin to seal the reinfiltration of pathogens. 

One of the difficulties is how to confirm the clinical vitality of pulp. Histological examination can verify the vitality of dental pulp, but is not practical for clinicians, who are limited to clinical and radiographic evaluations, which do not provide an accurate evaluation of pulp vitality. For this reason, more sensitive methods and/or instruments need to be developed.

It is possible that pulp regeneration using autologous DSC might become a routine therapy after endodontic treatment. However, autologous DSC sources are limited. Several DSC banks have been established, and patients have started to cryopreserve their DSCs. Perhaps the most promising solution might be induced pluripotent stem cells (iPSCs), cells that have been artificially derived through stem cell gene transfer into an adult somatic cell [90, 91]. As compared to ES Cells, iPSCs can be used for autologous tissue regeneration. Currently, transfection methods are retroviral based, which can induce unwanted health-related problems such as cancer, although many groups are working to develop new methods for gene delivery that are not retroviral based, such as protein or chemical induction. To date, no published reports of induced dental stem (iDS) cells have yet been reported, although iPSCs eventually may become the ultimate solution for cells source of pulp regeneration. 

## 5. Summary

It has been widely accepted that maintaining and regenerating dental pulp vitality is critical for long-term tooth viability. When any vital pulp remains, complete pulp regeneration and revascularization can be achieved after successful vital pulp therapy. However, as elucidated above, many issues must first be addressed and resolved before it will be possible to fully regenerate dental pulp de novo, or anew. At the present time, stem-cell-based tissue engineering approaches provide the most promising solution. Autologous dental pulp stem cells offer the best cell source but are not always available. The ability to successfully use iPSCs, and/or induced dental stem cells, for dental pulp regenerative therapies, could eventually provide a practical alternative cell source.
